# Syneresis‐Driven Self‐Refilling Printing of Geometry/Component‐Controlled Nano/Microstructures

**DOI:** 10.1002/advs.202405151

**Published:** 2024-08-29

**Authors:** Kota Shiba, Kayoko Saito, Kosuke Minami, Shunto Arai, Genki Yoshikawa, Luyi Sun, Mizuki Tenjimbayashi

**Affiliations:** ^1^ Research Center for Macromolecules and Biomaterials (RCMB) National Institute for Materials Science (NIMS) 1‐1 Namiki Tsukuba Ibaraki 305‐0044 Japan; ^2^ Materials Science and Engineering Graduate School of Pure and Applied Science University of Tsukuba 1‐1‐1 Tennodai Tsukuba Ibaraki 305–8571 Japan; ^3^ Polymer Program Institute of Materials Science and Department of Chemical & Biomolecular Engineering University of Connecticut Storrs CT 06269 USA; ^4^ International Center for Materials Nanoarchitectonics (MANA) National Institute for Materials Science (NIMS) 1‐1 Namiki Tsukuba Ibaraki 305‐0044 Japan

**Keywords:** microstructure, nanostructure, polydimethylsiloxane, printing, syneresis, wrinkles

## Abstract

Nano/microfabrication is of fundamental importance both in scientific and industrial situations. There are, therefore, many attempts at realizing easier, quicker, and more precise fabrication of various structures; however, achieving this aim without a bulky and costly setup is still challenging. Here, we introduce a facile and versatile means of printing an ordered structure consisting of nanoscale stripes and more complicated geometries including pillars and wavy form with a lateral resolution of single micrometers. To this end, we prepare a polydimethylsiloxane (PDMS) slab with an oxygen plasma‐induced wrinkled surface where liquid PDMS exudes by syneresis. Since this liquid PDMS is automatically loaded, the printing is repeatable without inking. A substrate moderately wettable to the liquid PDMS as well as amount/property‐controlled syneresis is primarily important for the creation of well‐defined structures. Precisely controlling these conditions will make this method universally applicable to diverse substrates and liquids including suspensions.

## Graphical abstract

We demonstrate an easy and versatile means of printing an ordered nano/microstructure using liquid polydimethylsiloxane (PDMS) exuded from a PDMS slab whose surface is periodically wrinkled. This method allows to print various nano/microstructures including stripes, pillars, and wavy form without ink loading. Printing with molecular and even larger species such as nanoparticles is possible toward functional pattern fabrication.

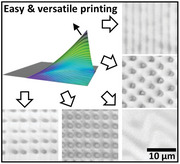

## 1 Introduction

Among various approaches including wet and dry processes, lithography is the one that has been conveniently used for nano/microstructure patterning everywhere in the world owing to its high reproducibility and preciseness.^[^ ^1^ ^]^ A wide variety of lithography‐based microfabrication methods have been developed so far, such as dip‐pen lithography (DPL),^[^ ^2^ ^]^ electron beam lithography (EBL),^[^ ^3^ ^]^ chemical lift‐off lithography (CLL),^[^ ^4^ ^]^ atomic force microscope (AFM)‐based cut‐and‐paste,^[^ ^5^ ^]^ photolithography (PL),^[^ ^6^ ^]^ nanoimprint lithography (NIL),^[^ ^7^ ^]^ and polymer pen lithography (PPL).^[^ ^8^ ^]^ These methods are divided into two groups: serial writing methods including DPL, EBL, CLL, and AFM‐based cut‐and‐paste, and parallel replication methods including PL, NIL, and PPL. The serial writing methods can create nano/microstructure patterns with high resolution. Furthermore, these methods can even write an arbitrary pattern using a scanning probe, while they require a bulky, expensive setup to precisely manipulate a probe that is usually a fragile cantilever except for EBL. As a result, the serial writing methods are costly and are limited in throughput. By contrast, the parallel replication methods including other related approaches such as various contact printing^[^ ^9^ ^]^ can achieve high‐throughput patterning over a wide area. These methods, however, can only duplicate a single pattern at a time as they use a stamp that has a predefined pattern on the surface. In addition, fabricating a predefined pattern is done by microfabrication that requires multiple steps in a clean environment. Another substantial limitation is “inking”. Unlike the serial writing approaches, some species that are supposed to be printed (i.e., ink) need to be carefully attached to the stamp surface in a controlled manner every time before printing. Although attempts have been made to solve the inking problems for easier and better operation,^[^ ^8b^ ^]^ loading an ink itself has always been a sensitive but inevitable step for this type of approach. If a simple, fast approach that can even overcome the challenging inking problem is developed for the patterning of nano/microstructures, such promising structures would become accessible to more and more people working in scientific and industrial fields, not only facilitating the use of them in existing applications but also finding a new way of using various complex surfaces.

**Scheme 1:**
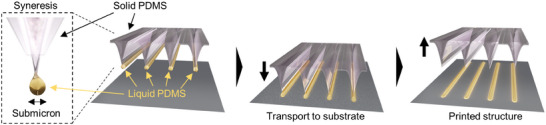
Printing of a nano/microstructure using syneresis‐based liquid PDMS exuded from solid PDMS whose surface has a periodic wrinkle structure.

In this paper, we describe a facile approach to printing an ordered structure consisting of stripes whose amplitude is in tens of nanometers while wavelength is in single micrometers. We accomplish this by depositing liquid polydimethylsiloxane (PDMS) exuded from a PDMS slab whose surface is periodically wrinkled due to an oxygen (O_2_) plasma treatment under stretching, as shown in **Scheme** **1**. Unlike the conventional printing techniques discussed above, this approach is free from inking; we intentionally prepare a soft PDMS that still contains much amount of PDMS base residues in the network structure so that it can work as a sufficient refill, allowing us to print the nano/microstructure repeatedly by syneresis without ink loading. The as‐printed structure is erasable, while it becomes very stable after being exposed to an argon (Ar) plasma for cross‐linking. In addition to the simple stripes, other types of structures such as pillars and wavy geometry are also printable through multiple printings using a viscous silicone oil‐added PDMS slab with various initial strains under the O_2_ plasma treatment. Furthermore, we discuss the potential of this approach toward functional patterns by demonstrating that nanoparticles can be printed together with PDMS.

## 2 Results and Discussion

**Figure 1:**
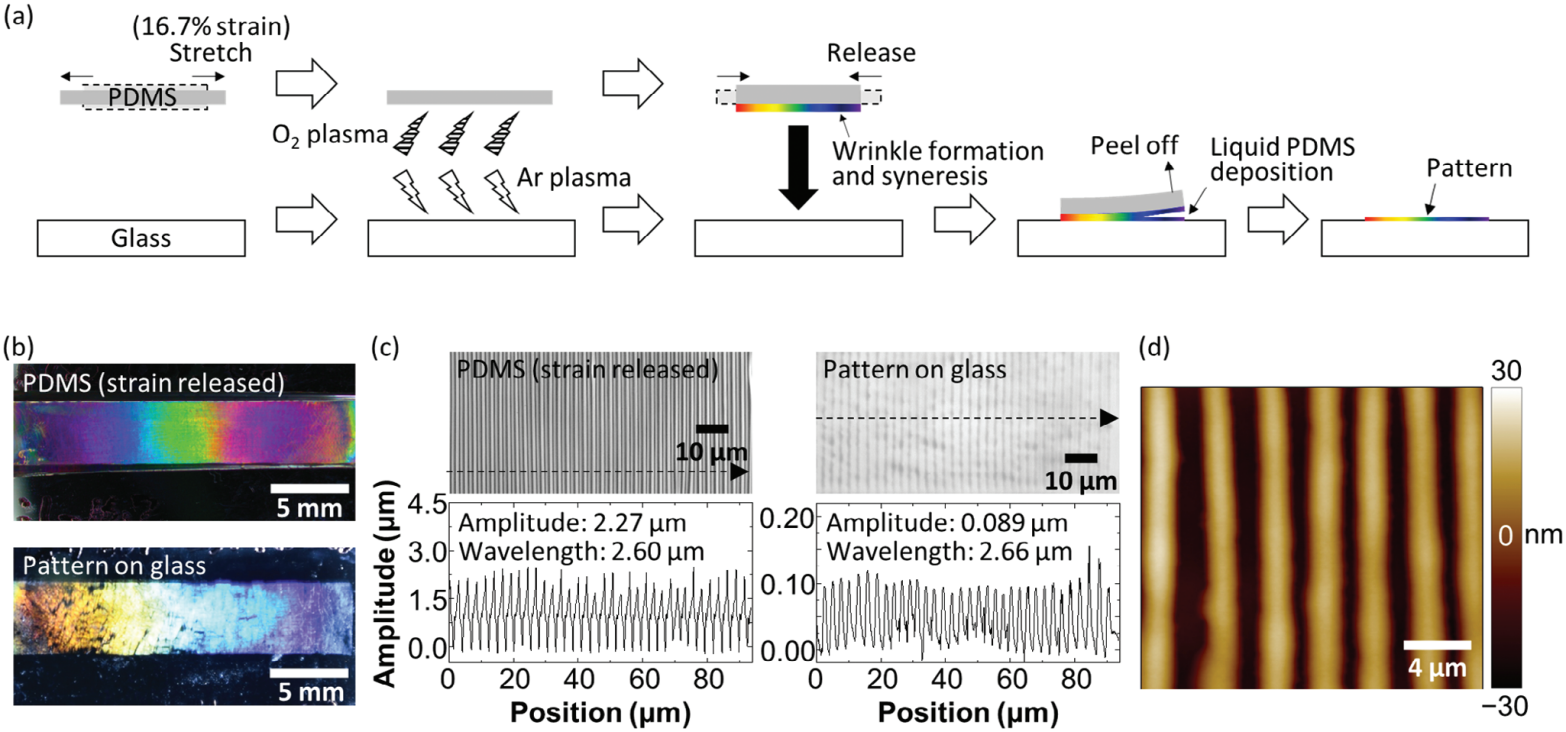
Overview of the present printing. a) A schematic of the printing procedure. b) Photos of the wrinkled PDMS (top) and the pattern printed on a glass surface (bottom). c) Laser microscope images and line profiles of the samples shown in (b). The line profiles are recorded along the dashed line arrow in each image. d) An AFM image of the pattern printed on a glass surface.

The present printing of a well‐ordered nano/microstructure is based on a phenomenon known as wrinkling. Wrinkles are formed in the skin when we squeeze the skin of our hand for example. Our skin consists of several layers where the thin, topmost layer is relatively stiffer than the bulk bottom layers. Since the stiff layer is more resistant to compression than the soft layer, periodic wrinkle formation occurs as a result of a buckling transition.^[^ ^10^ ^]^ The same wrinkling phenomenon can be observed in other material systems such as PDMS,^[^ ^11^ ^]^ and the concept of this study is to use the PDMS wrinkling for printing. For this purpose, we stretch a PDMS slab with a 16.7% strain and expose it to an O_2_ plasma under the stretching so that the stretched surface is oxidized to have a thin, glass‐like layer atop bulk PDMS, and wrinkles are formed accordingly by releasing the strain, as shown in **Figure** **1**a. We bring this PDMS into contact with an Ar plasma‐cleaned glass substrate and let the PDMS adhere to the glass for 1 h under constant pressure. Then, we find an iridescent pattern left on the glass surface after peeling off the PDMS which also shows iridescence, as shown in Figure 1b. Different contact times of up to 10 min lead to very light printings, whereas a 30 min printing results in a visible but still somewhat lighter printing than the one mentioned above, suggesting that 1 h is enough for a clear printing. Surface observations using a laser microscope and an AFMreveal that the pattern consists of a periodic array of wrinkles whose wavelength is 2.66 µm which is consistent with that of the wrinkles formed on the surface of the PDMS (2.60 µm), as shown in Figure 1c,d. Taking into account that the wrinkled PDMS is similar to a diffraction grating, the iridescent color depends on the wavelength of the wrinkles that is given as a function of the strain exerted on the PDMS, as experimentally shown in the literature.^[^ ^12^ ^]^

Apparently, the iridescent pattern originates from the PDMS wrinkles, but the question arising from this outcome is why and how the pattern is printed. It is syneresis that we focus on here to transfer the periodic structure existing on the PDMS surface. Syneresis is also a known phenomenon where a liquid is extracted or expelled from a gel.^[^ ^13^ ^]^ In the present case, something exuded from the PDMS surface is unreacted liquid PDMS remaining in the PDMS matrix, and we use it as ink for printing. Unlike a commonly used PDMS that is prepared by mixing a liquid PDMS base and a curing agent with a weight ratio of 10:1, we employ a weight ratio of 30:1, leaving more amount of PDMS base residues in the matrix. Considering that the 10:1‐PDMS contains ≈5 wt.% of the PDMS base,^[^ ^14^ ^]^ the 30:1‐PDMS should contain ≈15 wt.% or more, leading to easier syneresis. As already discussed, the iridescent pattern is printed using the 30:1‐PDMS, whereas almost nothing is left on a glass surface when we use the 10:1‐PDMS. Furthermore, this syneresis‐based printing is possible only if wrinkles are formed in the PDMS surface owing to the O_2_ plasma treatment under the stretching. Again, there is nothing left on a glass surface when we use an O_2_ plasma‐treated PDMS without being stretched (Figure S1, Supporting Information), indicating that squeezing plays a critical role in facilitating syneresis. To validate this idea, we also use an O_2_ plasma‐treated but unstretched PDMS that is left in an ambient condition for two weeks prior to the experiment so that we can confirm if spontaneous syneresis occurs. Consequently, nothing is printed, whereas a similar pattern is printed if this two‐weeks‐aged PDMS is stretched and treated with an O_2_ plasma in the same way as the fresh PDMS used to collect the results shown in Figure 1. Therefore, the principle of this printing is based on the facilitated syneresis on the wrinkled PDMS surface.

**Figure 2:**
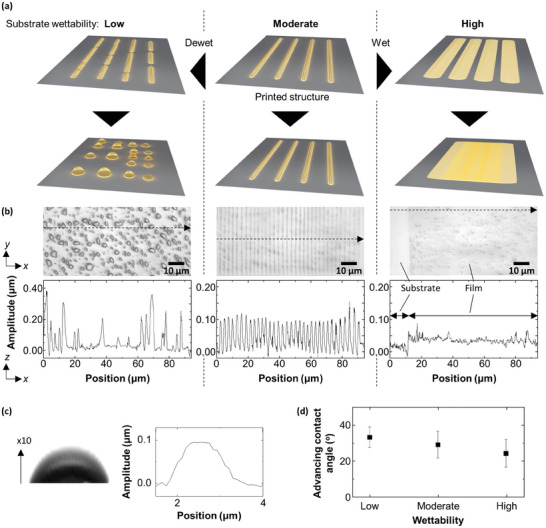
Proposed mechanism and verification. a) Substrate‐dependent change in the structure after printing. b) Laser microscope images and line profiles of the samples deposited on the three substrates shown in (a). The line profiles are recorded along the dashed line arrow in each image. c) A zoom‐up of a PDMS droplet deposited on a substrate with moderate wettability (left) and a line profile of a single wrinkle shown in the middle of (b) (right). Note that the left image is exaggerated in the z‐direction so that the scale is consistent with that of the line profile on the right. d) A plot of advancing contact angles measured for the three substrates where a PDMS droplet is deposited. All error bars represent the standard deviation.

Another essential factor that affects the printing is the wettability of a substrate. Since liquid PDMS exuded from the wrinkled surface is brought into full contact with a glass substrate, the morphology of the printed structure must depend on how the liquid PDMS spreads on the surface. On the basis of this assumption, we prepare three glass substrates with different wettability to liquid PDMS (denoted as low, moderate, and high wettability) and use them for printing. The ordered nano/microstructure should be printed only if the surface is moderately wettable to liquid PDMS, as illustrated in **Figure** **2**a. The liquid PDMS is expected to be nearly entirely spread on the high wettability surface while the liquid PDMS can easily dewet on the low wettability surface so that the printed stripe is split into polydispersed droplets. Actual experiments using the three substrates prove that these assumptions are correct, as shown in Figure 2b. Moreover, we find that the contact angle of a PDMS droplet deposited on the moderate‐wettability glass measured an hour after deposition is ≈10°, which is in excellent agreement with that of a cured PDMS wrinkle, as shown in Figure 2c. This contact angle is different from those measured for a PDMS droplet deposited on the high and low wettability glass: 5° and 13°, respectively (Figure S2, Supporting Information). Note that here we use a PDMS droplet to measure a contact angle instead of using a water droplet by following a standard protocol because of the necessity to characterize the specific affinity between PDMS and the substrates. Considering that the liquid PDMS is pushed to spread in the printing process, an advancing contact angle−the threshold angle of liquid spreading−should also closely correlate with the present results. The advancing contact angles measured for the high, moderate, and low wettability glasses are 24°, 29°, and 33°, respectively, meaning that this trend is consistent with the aforementioned trend for the static contact angles, as shown in Figure 2d. Taking account of all the results discussed above, it is clear that wettability is the primary factor influencing the printing process.

**Figure 3:**
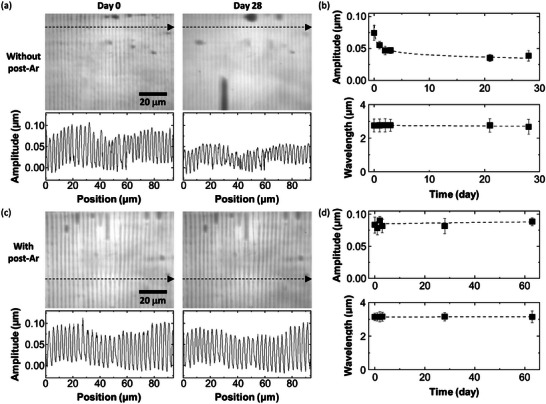
Time‐dependent change in morphology of the printed pattern with and without an Ar plasma post‐treatment. a) Laser microscope images and line profiles of the pattern right after printing (left) and 28 days after printing (right). b) Plots of amplitude (top) and wavelength (bottom) of the samples shown in (a) as a function of time (day). c) Laser microscope images and line profiles of the Ar plasma post‐treated pattern right after printing (left) and 28 days after printing (right). d) Plots of amplitude (top) and wavelength (bottom) of the samples shown in (c) as a function of time (day). All the line profiles are recorded along the dashed line arrow in each image. All error bars represent the standard deviation.

Since we use syneresis for printing, the as‐deposited PDMS is most probably in a liquid state, and thus, it can easily undergo some changes in morphology over time in ambient conditions. In an actual experiment, the printed pattern seems to gradually fade away; the contrast of the printed structure becomes lighter, as shown in **Figure** **3**a,b. The amplitude of the wrinkles is estimated to be ≈0.080 µm right after deposition, but it becomes almost half after 28 days while the wavelength does not change at all (see also Figure S3, Supporting Information). By contrast, we notice that several regions of the printed structure conversely become darker over time, meaning that the amplitude increases (Figure S4, Supporting Information). There is also a spot that appears and becomes darker during the first few days, but then it becomes lighter until 28 days. What these phenomena indicate is that the printed structure is indeed liquid and unstably movable on the glass with moderate wettability that is hydrophilic and does not favor hydrophobic PDMS accordingly. However, an Ar plasma treatment after printing can stabilize the printed structure. An Ar plasma for 10 s at 25 W is sufficient to obtain a stable structure whose morphology rarely changes even after two months, as shown in Figure 3c,d (see also Figure S5, Supporting Information). The reason why this occurs is that the Ar plasma treatment facilitates the cross‐linking of the deposited PDMS,^[^ ^15^ ^]^ resulting in the formation of stable PDMS that is in a solid state.

**Figure 4:**
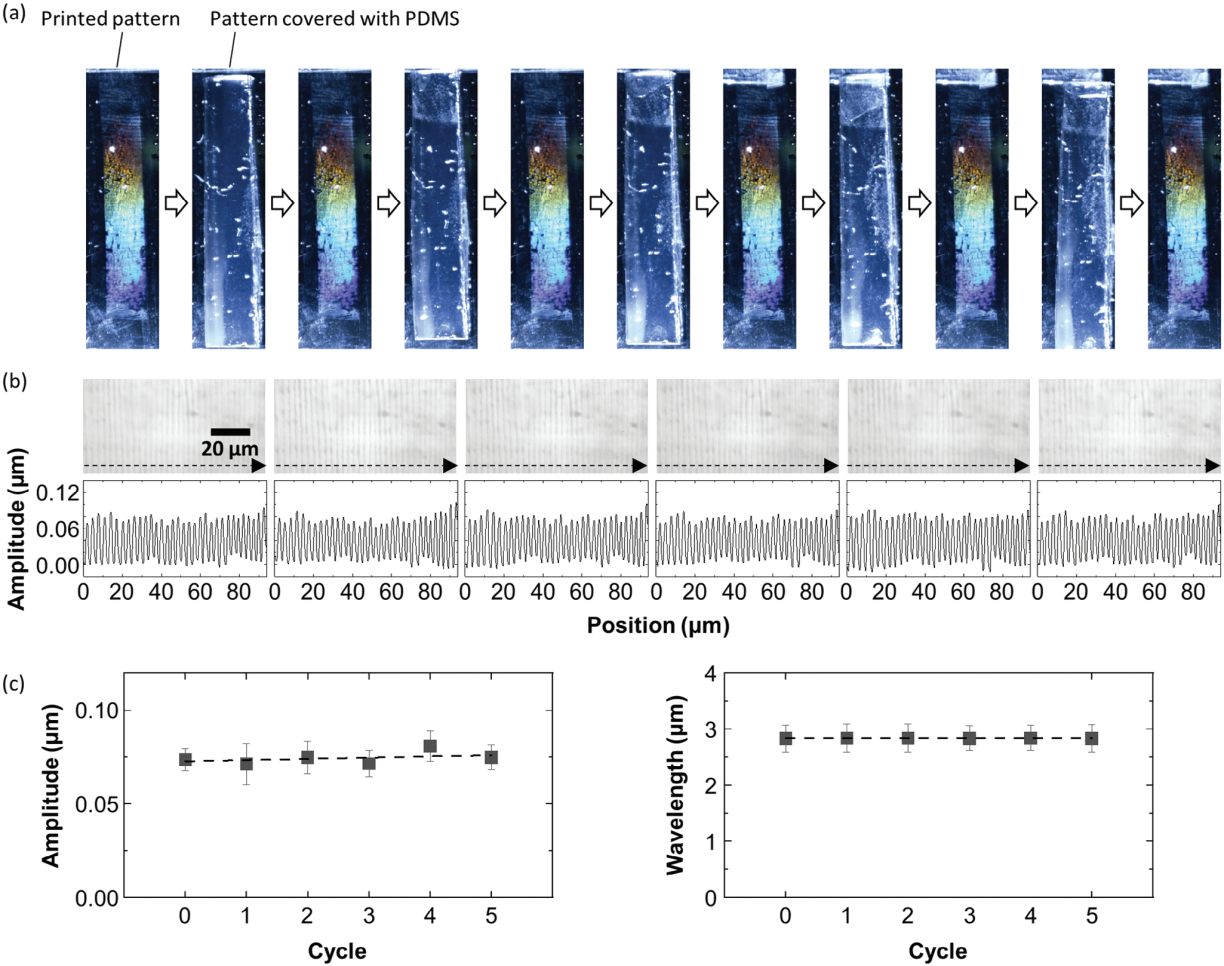
PDMS‐based tape test to evaluate the robustness of the Ar plasma post‐treated pattern. a) Photos of the Ar plasma post‐treated pattern before and after the robustness test. A pristine PDMS slab is brought into contact with the printed pattern and then is peeled off. This cycle is repeated up to five times, and the surface is observed using a laser microscope right after each cycle. b) Laser microscope images and line profiles of the samples shown in (a). The line profiles are recorded along the dashed line arrow in each image. c) Plots of amplitude (left) and wavelength (right) of the sample shown in (a) as a function of the number of cycles. All error bars represent the standard deviation.

The printed PDMS with the Ar plasma post‐treatment is mechanically robust. We bring a pristine 30:1‐PDMS slab into contact with the patterned glass and peel it off, as shown in **Figure** **4**a. As expected, when the glass is covered with the PDMS slab, an iridescent color that is due to the printed pattern completely disappears. Lacking a contrast in refractive index between the PDMS slab and the printed PDMS can explain the disappearance in iridescence. No air pocket is formed at the interface owing to the flexibility of the 30:1‐PDMS and the rather flat printed structure that allows for close contact between the PDMS slab and the patterned substrate (Figure S6, Supporting Information). Then, the color appears again without any change in appearance by removing the adhered PDMS. We observe the printed pattern at each step of the adhering‐removing cycles using a laser microscope and confirm that there is almost no change both in amplitude and wavelength even after repeating the cycle multiple times, as shown in Figure 4b,c. The same disappearance in iridescence is also observed by using a stiffer 5:1‐PDMS (Figure S7, Supporting Information). On the contrary, if we cover the printed pattern using another glass substrate, the iridescent color is clearly observable through the top glass because of the poor contact that leaves air between the two glass plates. These results show the robustness of the structure as well as the switchable iridescence that is an interesting feature of the printed pattern.

**Figure 5:**
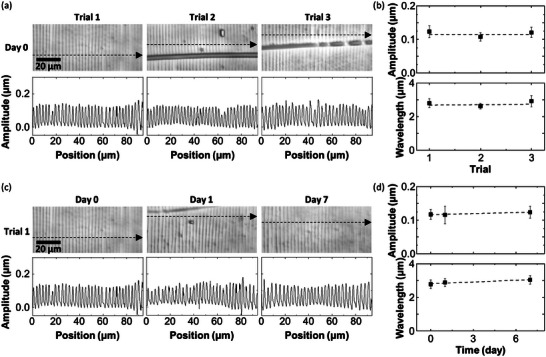
Repeated printing using the same PDMS. a) Laser microscope images and line profiles of the pattern printed using the same PDMS: first trial (left), second trial (middle), and third trial (right). b) Plots of amplitude (top) and wavelength (bottom) of the samples shown in (a) as a function of the number of trials. c) Laser microscope images and line profiles of the pattern printed using the same PDMS: printed again 1 day (middle) and 7 days (right) after the first printing (left) that is also shown in the left of (a). d) Plots of amplitude (top) and wavelength (bottom) of the samples shown in (c) as a function of time after PDMS preparation (day). All the line profiles are recorded along the dashed line arrow in each image. All error bars represent the standard deviation.

A great advantage of using syneresis is that we no longer need to carefully ink every time we print a pattern. To prove this, we repeat a printing multiple times using the same PDMS without any additional treatment between each printing. Specifically, three independent patterns are printed on the first day using the same PDMS without additional O_2_ plasma under stretching between each printing, resulting in very similar patterns in terms of amplitude and wavelength, as shown in **Figure** **5**a,b. These reproducible printings suggest that the amount of exuded PDMS is consistent. A little bit bolder lateral lines which are due to fractures on the surface of a PDMS slab are observed in the second trial than in the first trial, while the amplitude and wavelength are still almost consistent. No significant increase of the lateral lines is recognized after the second trial so the printing becomes far less variable after a PDMS slab is used for printing even once. Another printing is also made 24 h after the first three trials, and there is almost no variation. Surprisingly, we confirm that the pattern is still duplicated even after a week of using the same PDMS, as shown in Figure 5c,d. An important finding indicated by the fact that the printings are almost identical is that the amount of exuded PDMS does not change significantly over time after 1 h, as discussed earlier by focusing on contact time for printing. Given that the amount of exuded PDMS depends on the swelling of a solid PDMS used for printing and that the actual amount of exuded PDMS deposited on the substrate depends on several parameters such as viscoelasticity of the exuded PDMS, peeling‐off speed, contact time, and wettability, the reproducible printing should be due to the consistency in the above‐mentioned parameters in a series of experiments. Considering that 15 wt.% or more amount of liquid PDMS residues are present in the swollen 30:1‐PDMS, we can theoretically repeat the similar printing several thousand times or even more using the same piece of PDMS (see Text S1 and Table S1, Supporting Information for details).

To extend the potential of syneresis‐based printing, we also print a pattern that is composed of not only PDMS but additional species. For this purpose, we select one of the silicone oils that is miscible with the liquid PDMS base resin and is more viscous than the PDMS base resin so that the printed pattern can not only be functionalized with the silicone oil but also be mechanically less movable due to the high viscosity. The silicone oil containing PDMS (denoted as oil‐PDMS) looks exactly the same as the pure PDMS in terms of transparency, but a tensile test reveals that the Young's modulus of the oil‐PDMS is ≈80 kPa while that of the pure PDMS is ≈120 kPa, suggesting that the two components are homogeneously mixed together and the oil exists in the PDMS matrix. According to a previous report, various silicone oil‐PDMS systems show syneresis,^[^ ^16^ ^]^ meaning that oils are not cross‐linked with PDMS but are present in the matrix without chemical constraints. After experimentally confirming that syneresis certainly occurs in the present material combination (Figure S8, Supporting Information), we use the oil‐PDMS for printing and find that an ordered wrinkle structure is formed on the glass with moderate wettability same as the case of the pure PDMS (Figure S9, Supporting Information). We find that the wavelength of the pattern printed using the oil‐PDMS is larger than that of the pattern printed using the pure PDMS (3.49 and 2.92 µm, respectively). Considering that the oil‐PDMS is softer than the pure PDMS as discussed, the difference in wavelength should be reasonable because the trend where a smaller Young's modulus gives a larger wavelength is consistent with the one obtained by analytical calculations reported previously.^[^ ^17^ ^]^ Another important difference here is that the printed structure is more robust than that obtained by using the pure PDMS; the as‐printed wrinkle structure does not drastically change over time even without an Ar plasma post‐treatment. Thus, the printed structure should be a mixture of the viscous oil and PDMS as expected.

**Figure 6:**
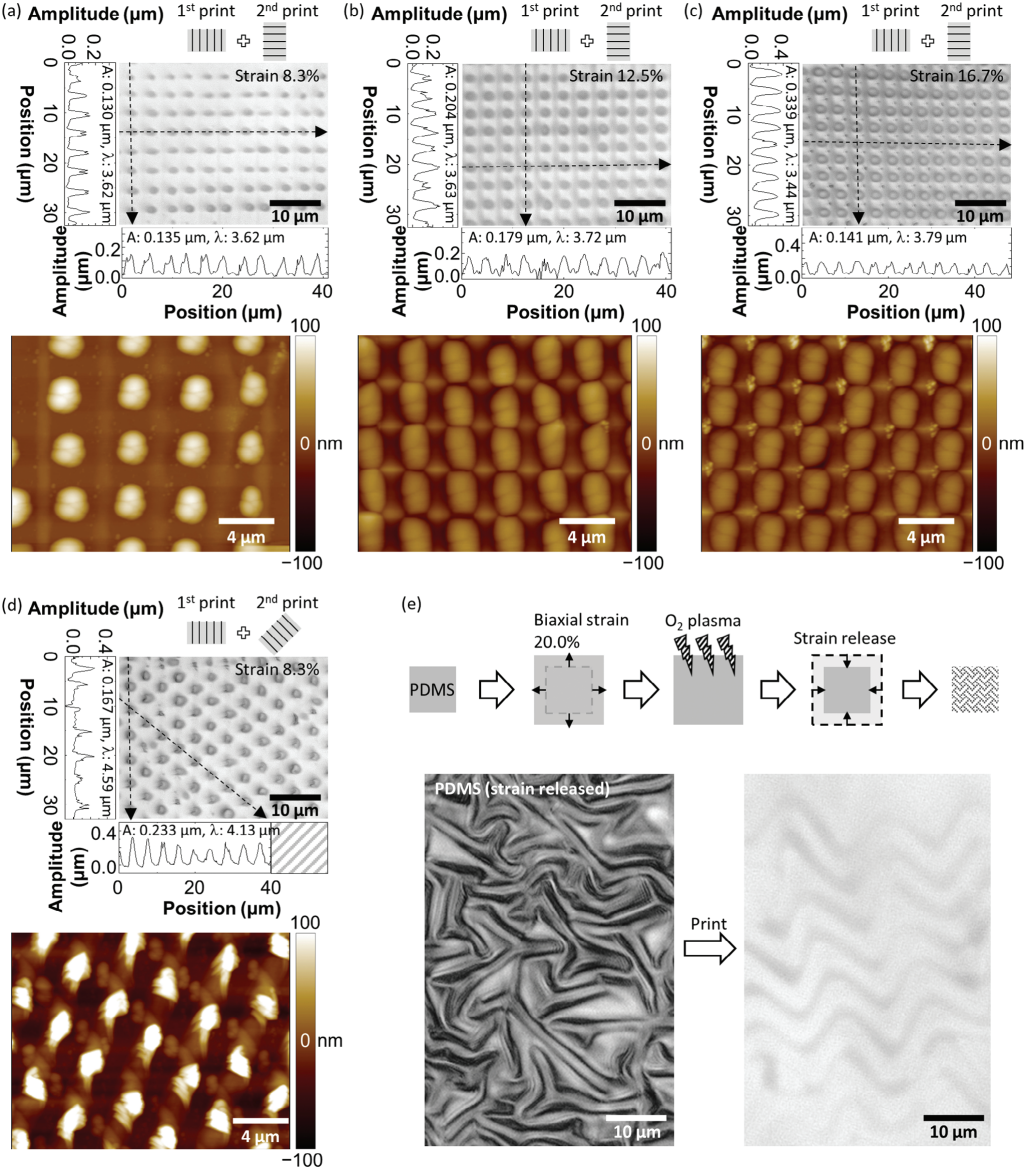
Demonstration of various complex patterning. a–d) Laser microscope images and AFM images of the dually printed pattern (either 90° or 45° rotation at the second printing). The strain applied to an oil‐PDMS slab under the Ar plasma treatment is 8.3% for (a) and (d), 12.5% for (b), and 16.7% for (c). The line profiles recorded along the dashed line arrows in the image are also shown. “A” and “λ” are the amplitude and the wavelength of the printed structure, respectively. e) Laser microscope images of the biaxially stretched PDMS (left) and the printed pattern using the biaxially stretched PDMS (right). The experimental procedure for preparing the biaxially stretched PDMS is schematically shown above.

In addition to the simple structure consisting of the straight wrinkles already discussed, we demonstrate by using the oil‐PDMS that various complex structures can be printed on a substrate with moderate wettability. We do this by repeating the printing twice on the same substrate but with 90° or 45° rotation at the second printing. This two‐way printing results in an ordered pillar‐like structure, as shown in **Figure** **6**a–d. The amplitude of the pillars increases from 0.13 to 0.34 µm as the initial strain increases from 8.3% to 16.7%. This increase in amplitude is due to the greater amount of liquid PDMS squeezed out of the PDMS matrix by syneresis with more stretching. Importantly, the PDMS with an 8.3% strain requires at least a 15 min interval before each printing step; printing without this interval results in a fairly faint pattern. By contrast, the other two PDMS can be used for another printing soon after the previous printing, meaning that a sufficient amount of liquid PDMS is loaded immediately. Although the wavelength does not significantly vary among the three patterns obtained with the different strains (≈3.5 µm), the pattern obtained with the 45° rotation shows a larger wavelength of ≈4.6 µm that is close to a geometrically expected value of 4.9 µm. What is remarkable here is that the structure becomes more complex as the strain increases; the original wrinkles are still present between the pillars, especially in the case of the initial strain of 12.5% and 16.7%. Thus, the resultant structure is, to be more precise, an alternate array of vertical lines and pillars. A possible reason for the formation of pillars instead of lateral lines at the second printing is probably that the liquid PDMS exuded along the PDMS wrinkle is evenly segmented by the orthogonally printed stripes. At this stage, some amount of PDMS that forms the stripe structure might be dissolved in the liquid PDMS being pressurized for a certain period. In addition, the formation of the pillars must be affected by the surface states of the glass substrate and PDMS that determine how their surfaces get wet/dewet with the liquid PDMS. Another demonstration of the complex structure is a wavy pattern printed using a biaxially stretched PDMS, as shown in Figure 6e. In this case, a PDMS slab is stretched vertically and laterally at the same time with a 20.0% strain, and the biaxial strain is released after an O_2_ plasma treatment. Consequently, the wavy structure formed on the surface of the PDMS is successfully printed on a glass surface, the same as other complex structures discussed above.

**Figure 7:**
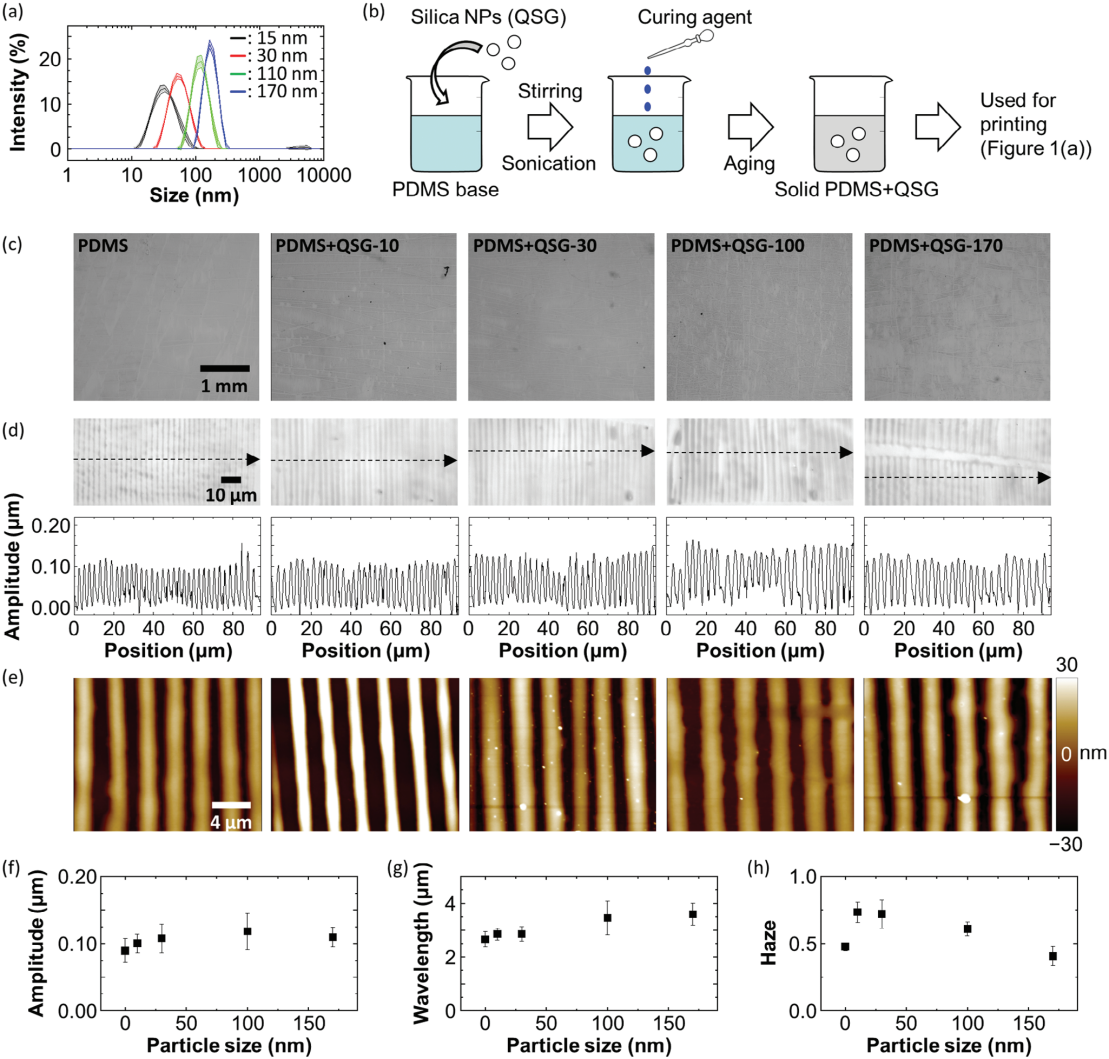
Multicomponent printing using size‐controlled nanoparticles. a) Particle size distributions of the commercial silica nanoparticles (QSG series) measured by DLS. The disclosed average particle sizes of QSG‐10, QSG‐30, QSG‐100, and QSG‐170 are 15, 30, 110, and 170 nm, respectively. b) A schematic of the preparation of silica nanoparticles dispersed in a solid PDMS. c–e) Low‐magnification laser microscope images (c), laser microscope images and line profiles (d), and AFM images (e) of the printed pattern that is a mixture of PDMS and silica nanoparticles. The line profiles in (d) are recorded along the dashed line arrow in each image. f–h) Plots of amplitude (f), wavelength (g), and haze values (h) of the samples as a function of particle size. All error bars represent the standard deviation.

To demonstrate another promising aspect of the present approach, we use hydrophobic silica nanoparticles with four different sizes (QSG‐10, QSG‐30, QSG‐100, and QSG‐170) as an additive for printing. The average sizes of them are 15, 30, 110, and 170 nm according to the data sheet. Dynamic light scattering (DLS) measurements reveal that the peak positions for QSG‐100 and QSG‐170 are almost identical to the average sizes mentioned above whereas QSG‐10 and QSG‐30 show relatively broad size distributions whose peak positions are slightly larger than their disclosed average sizes, as shown in **Figure** **7**a. This is due to aggregation of the hydrophobic nanoparticles in a well water‐miscible medium, isopropanol, used for the measurements. Owing to the hydrophobic surface of the nanoparticles, they are fairly easily mixed with the liquid PDMS base by the procedure schematically shown in Figure 7b. Regardless of the rather thick concentration of 3.3:1 (PDMS:QSG by weight), no visible aggregates are observed on the bottom. The Young's moduli of the nanoparticles‐PDMS are 220, 260, 200, and 170 kPa for QSG‐10, QSG‐30, QSG‐100, and QSG‐170, respectively. Taking account of the amount of hard nanoparticles in the soft PDMS matrix, these values seem to be rather small. There is only a slight difference between the Young's modulus of the pristine PDMS (120 kPa) and the aforementioned values, suggesting that the added nanoparticles and PDMS weakly interact with each other so that the nanoparticles are expected to be printed by syneresis.

We show experimental verification that the printing with nanoparticles is possible. The printed patterns look similar in terms of their appearance and periodic structures except for QSG‐100 and QSG‐170 that give relatively rough patterns with larger wavelength values than the others, as shown in Figure 7c–g. The AFM images reveal that the PDMS structures co‐printed with QSG‐30, 100, and 170 have multiple bumps that should be due to embedded silica nanoparticles, as shown in Figure 7e. Importantly, the printed patterns for the pristine PDMS and the two nanoparticles (QSG‐10 and QSG‐30) are almost identical, but the haze values measured for these samples are clearly different, as shown in Figure 7h. Since a haze value is given as a difference between a total transmittance and a parallel transmittance divided by the total transmittance, this basically suggests that a haze value is an indicator of how much light scattering occurs in a transparent sample. Given that the printed patterns originated from QSG‐10 and QSG‐30 give haze values of ≈0.75 that is 1.5 times higher than that for the pristine PDMS‐based pattern, there is apparently extra light scattering occurring there in the printed patterns, most probably due to the presence of the nanoparticles. The exudence of the nanoparticles from the PDMS is supported by previous researches that report a typical PDMS has a mesh‐like structure whose mesh size ranges around a few tens of nanometers and ≈100 nm depending on the swollen state.^[^ ^18^ ^]^ Therefore, we expect that nanoparticles with a size up to at least 170 nm can be co‐printed with liquid PDMS by the present approach.

## 3 Conclusion

We show a facile approach to printing a well‐defined nano/microstructure on a glass substrate using a PDMS slab that has an ordered wrinkle structure on its surface created with an O_2_ plasma under stretching. This printing method is based on the syneresis of liquid PDMS from the wrinkled PDMS surface onto the substrate whose surface wettability is properly controlled to prevent the printed PDMS pattern from collapsing before being stabilized by an Ar plasma post‐treatment for cross‐linking.

In addition to a few parameters focused on in this paper including the wettability and the initial strain exerted on the PDMS under the O_2_ plasma treatment to induce the wrinkling, a lot of others will affect the quality and variety of printed patterns; the wavelength depends not only on the stiffness of PDMS but also various conditions of the plasma treatment such as power, pressure, and duration.^[^ ^19^ ^]^ Since the amplitude should be influenced by the amount of the liquid PDMS exuded from a PDMS slab, the initial strain and the stiffness of the PDMS would be also critical factors to be explored toward precisely controlled printing. Moreover, the remarkable ability to print additives together with the liquid PDMS allows for tuning not only surface properties but also physical properties including viscoelasticity. This could help fine‐tune a printed structure by manipulating liquid bridging and wetting‐dewetting behavior^[^ ^20^ ^]^ toward the one with complicated morphologies such as serif‐T‐shaped structures that show superomniphobicity^[^ ^21^ ^]^ or an array of assembled chiplets that can be used for practical display applications.^[^ ^22^ ^]^ Considering that the present printing requires a glass surface that is carefully treated to show a liquid PDMS contact angle of ≈30° while other substrates probably require a surface pre‐treatment to realize an optimum wettability for printing, tunable viscoelasticity would also help achieve consistent printing without being bothered very much by a possible variation in wettability that might accidentally occur. Furthermore, using mucin or some other species that show spinnability would be useful for freely constructing various 3D structures, opening up access to highly‐designed geometries toward bioinspired hierarchical structures^[^ ^23^ ^]^ and metasurfaces.^[^ ^24^ ^]^ Twisting or any arbitrary movement during the peeling off step in combination with Ar plasma curing might work as a new type of 3D printing technique toward complex 3D structures. Co‐printing with even larger species such as various nanoparticles will also lead to the formation of diverse hierarchical structures that exist in nature in the form of, for example, rice leaves that realize directional water harvesting.^[^ ^25^ ^]^ Multiscale additives ranging from nano to macro that are potentially applicable to the present approach can play a significant role in mimicking and building various hierarchical structures observed in natural species. Finally, another critical feature where inking is no longer necessary will be of great importance when this method is used to print a variety of aforementioned functional patterns in mass production.

## 4 Experimental Section

### Preparation of PDMS

A kit including PDMS base and curing agent (Sylgard 184, DowCorning) was used to prepare PDMS slabs. The liquid PDMS mixture, consisting of the base and the curing agent with a weight ratio of 30:1, was degassed and poured into a container made of polystyrene. After curing at 65 °C for 24 h, the PDMS was cut into pieces for further experiments.

For multicomponent printing, the aforementioned recipe for PDMS preparation was modified; silicone oil (KF‐96H‐60,000cs, Shin‐Etsu Chemical Co., Ltd.) or silica nanoparticles (QSG‐10, QSG‐30, QSG‐100, and QSG‐170, Shin‐Etsu Chemical Co., Ltd.) was added to the PDMS base with a weight ratio of 5:1 (base:silicone oil), or 3.3:1 (base:silica nanoparticles), respectively. In the case of the QSG series, the silica nanoparticles were slowly added to the PDMS base under stirring. The PDMS/oil or PDMS/nanoparticle mixture was then mixed with the curing agent with a weight ratio of 30:1 (base:curing) after confirming there were no visible aggregates on the bottom. After curing at 65 °C for 24 h, two types of the binary PDMS were cut into pieces for further experiments, the same as the pristine PDMS.

### Pattern Printing on a Glass Substrate

The whole procedure is schematically shown in Figure 1a. A PDMS slab (10 mm × 35 mm × 2.7 mm) was stretched with 16.7% strain and was exposed to an O_2_ plasma using a low‐pressure plasma system (Femto, version B, Diener Electronic GmbH + Co. KG.) under the stretching. The conditions for the plasma treatment were 60 W, 0.6 mbar, and 60 s. Then, the strain was slowly but completely released so that well‐defined wrinkles were formed on the surface. The wrinkled PDMS was brought into contact with a glass substrate whose surface was exposed to an Ar plasma (100 W, 0.6 mbar, 1 s) beforehand. To minimize the variance in the amount of exuded liquid PDMS, the above‐mentioned contact process should be done right after the plasma treatment. The PDMS‐attached glass was placed under constant pressure using a 250 g weight for 1 h. Finally, the PDMS was carefully peeled off from the substrate, leaving a pattern on its surface. Another Ar plasma treatment (25 W, 0.6 mbar, 10 s) was performed to cure and stabilize the printed pattern. The same printing protocol was also conducted using the following two glass substrates that had different wettability to liquid PDMS: low wettability glass (a regular slide glass, product code: 110 201, Muto Pure Chemicals Co., Ltd.) and high wettability glass (modified with phenylsilane^[^ ^26^ ^]^).

For other types of printed structures consisting of pillars, the same printing protocol was repeated twice using the oil‐PDMS at the same position but with a 90° or 45° rotation at the second printing. The duration for each step—first and second printing—was 30 min, and the second printing was performed soon after the Ar plasma treatment using the same piece of PDMS as the first printing without additional O_2_ plasma treatment. Three PDMS slabs with different initial strains (8.3%, 12.5%, and 16.7%) were used for this printing. Note that there was an interval of 15 min between the strain release after an O_2_ plasma treatment and the beginning of printing to facilitate syneresis only if the PDMS with an 8.3% strain was used. The other two PDMS slabs were used for printing without any interval. A wavy structure was printed using a PDMS slab (35 mm × 35 mm × 2.5 mm) that was biaxially stretched with a 20.0% strain under the O_2_ plasma treatment, as schematically shown in Figure 6e.

### Characterization

The wrinkled PDMS and printed patterns were observed by using a 3D surface profiler (VK‐X3000, KEYENCE Corporation, Japan) under the laser confocal mode. The height profiles were measured by tapping‐mode AFM (MFP‐3D Origin AFM, Asylum Research, UK). Contact angles were measured using a contact angle meter (Drop Master‐SA‐Cs1, Kyowa Interface Science Co., Ltd., Japan). The volume of the probe liquid (ultrapure water or liquid PDMS) was 5 µL. Ultrapure water with 18.2 MΩ/cm resistance was obtained using a Direct‐Q UV3 system (Merck KGaA, Germany). Sizes of silica nanoparticles were measured using DLS (Zetasizer Nano ZSP, Malvern Panalytical Ltd., UK) equipped with a He‐Ne laser operating at 4 mW power and 633 nm wavelength, and a computer‐controlled correlator at a 173° accumulation angle. Haze values—the ratio of backlight diffusion over total transmittance—were measured using a haze meter (NDH7000SP2, Nippon Denshoku Industries Co. Ltd., Japan). The light source was a white LED.

## Conflict of Interest

The authors declare no conflict of interest.

## Supporting information

Supporting Information

## Data Availability

The data that support the findings of this study are available from the corresponding author upon reasonable request.
