# Supplementary material for: Syneresis‐Driven Self‐Refilling Printing of Geometry/Component‐Controlled Nano/Microstructures
Source: Adv Sci (Weinh). 2024 Aug 29;11(40):2405151. doi: 10.1002/advs.202405151 (PMC11515907; doi:10.1002/advs.202405151)
Supplement: Supplementary file 1 — Supporting Information [file ADVS-11-2405151-s001.docx]

Supporting Information

Syneresis-Driven Self-Refilling Printing of Geometry/Component-Controlled Nano/Microstructures

Kota Shiba*, Kayoko Saito, Kosuke Minami, Shunto Arai, Genki Yoshikawa, Luyi Sun, and Mizuki Tenjimbayashi


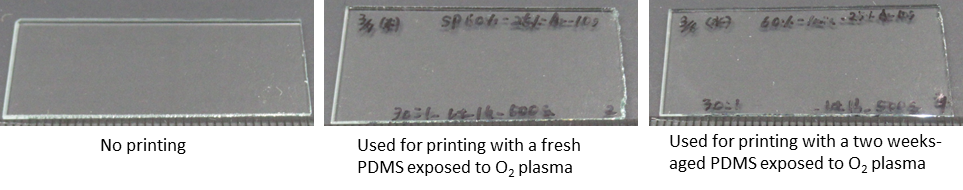


**Figure S1.** Photos of glass substrates: no printing (left), used for printing with a fresh PDMS exposed to O_2_ plasma (middle), and used for printing with a two weeks-aged PDMS exposed to O_2_ plasma (right).


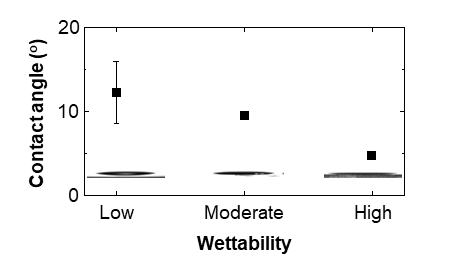


**Figure S2.** A plot of contact angles measured for a PDMS droplet deposited on the three substrates. The inset images show actual PDMS droplets used to measure the contact angles.


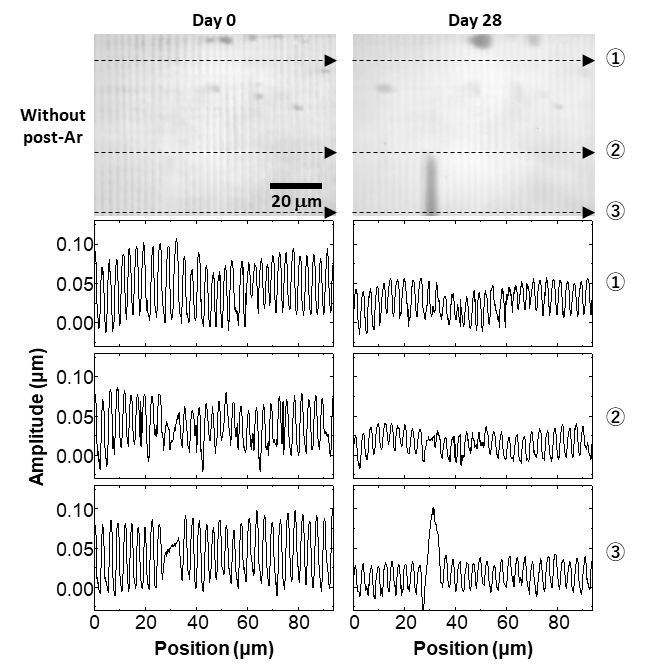


**Figure S3.** Laser microscope images and line profiles of the printed patterns without an Ar plasma post-treatment: right after printing (left) and 28 days after printing (right).


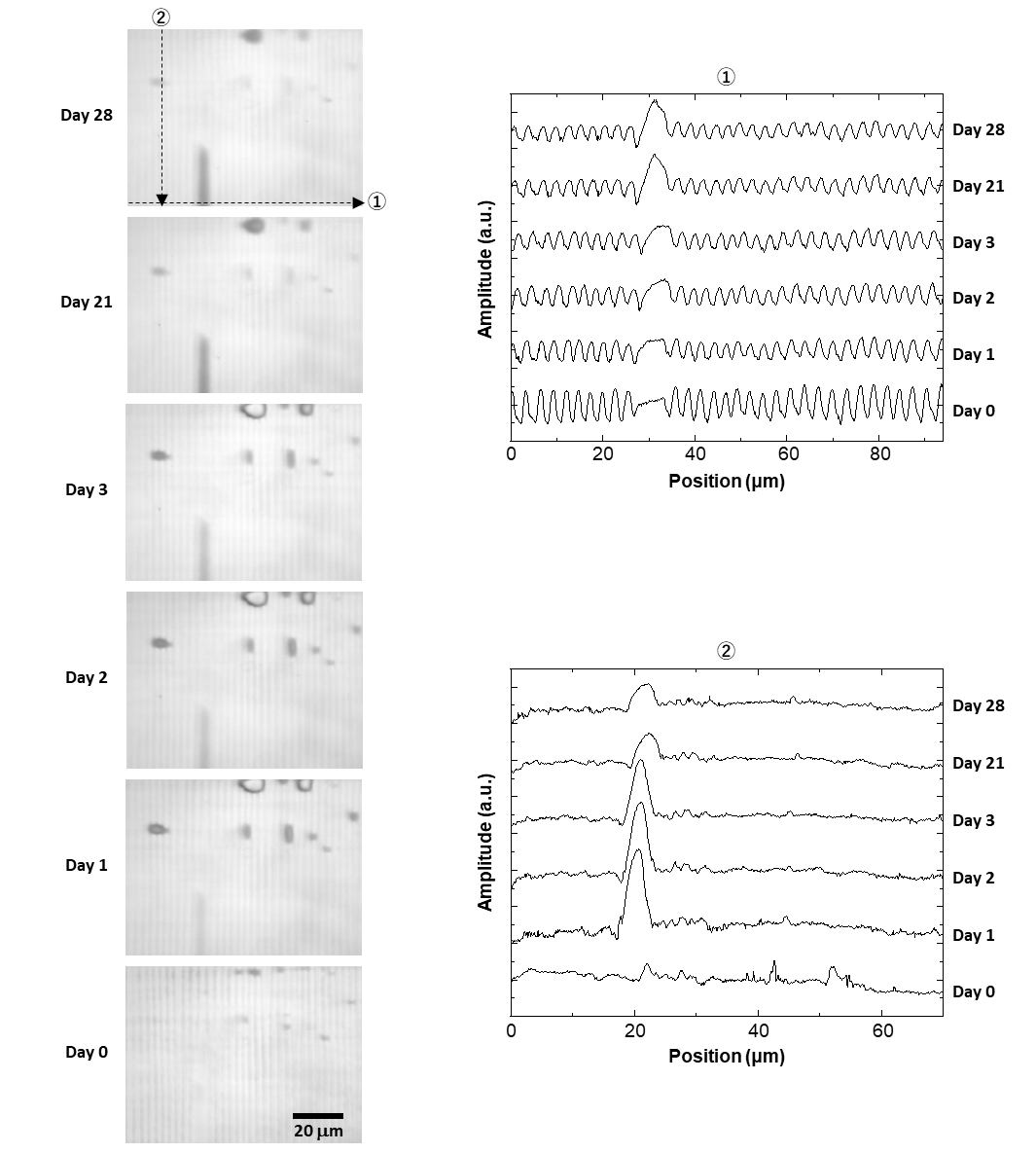


**Figure S4.** Laser microscope images of the pattern shown in Figure 3(a) right after printing to 28 days after printing (from the bottom to the top). The line profiles recorded along the two dashed line arrows in the top image are also shown on the right.


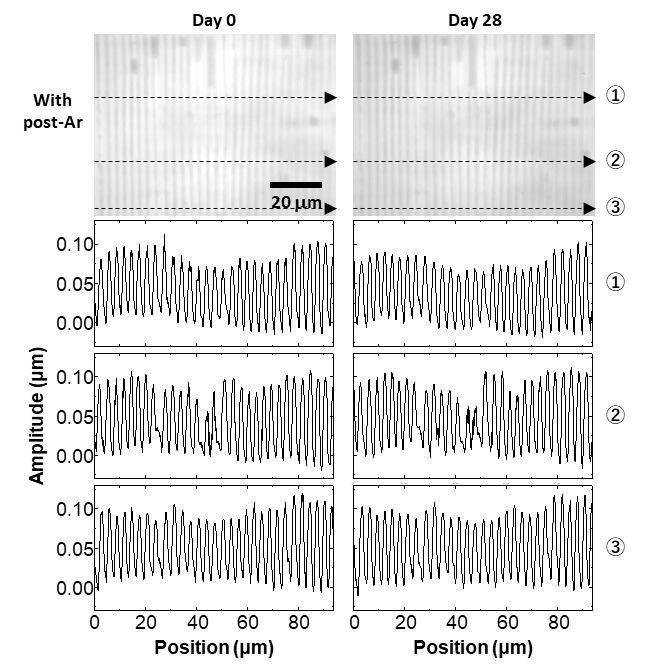


**Figure S5.** Laser microscope images and line profiles of the printed patterns with an Ar plasma post-treatment: right after printing (left) and 28 days after printing (right).


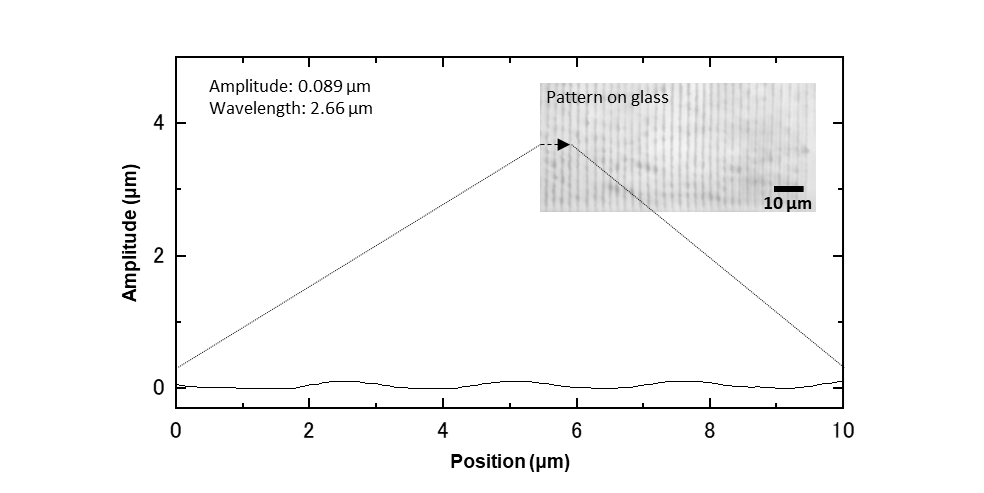


**Figure S6.** A line profile of the sample shown in Figure 1(c). This profile is recorded along the dashed line arrow that is shown in the inset image.


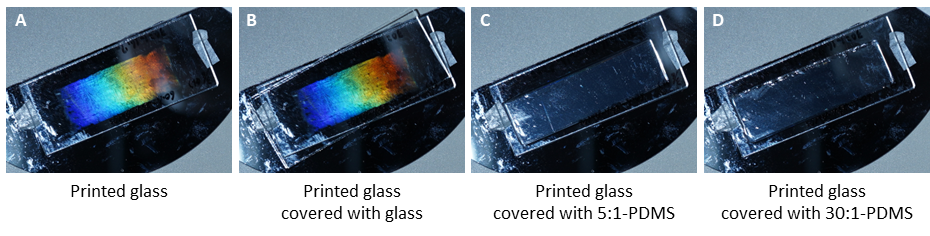


**Figure S7.** Photos of a printed pattern with/without a cover. A: no cover, B: covered with a glass, C: covered with a 5:1-PDMS, and D: covered with a 30:1-PDMS.


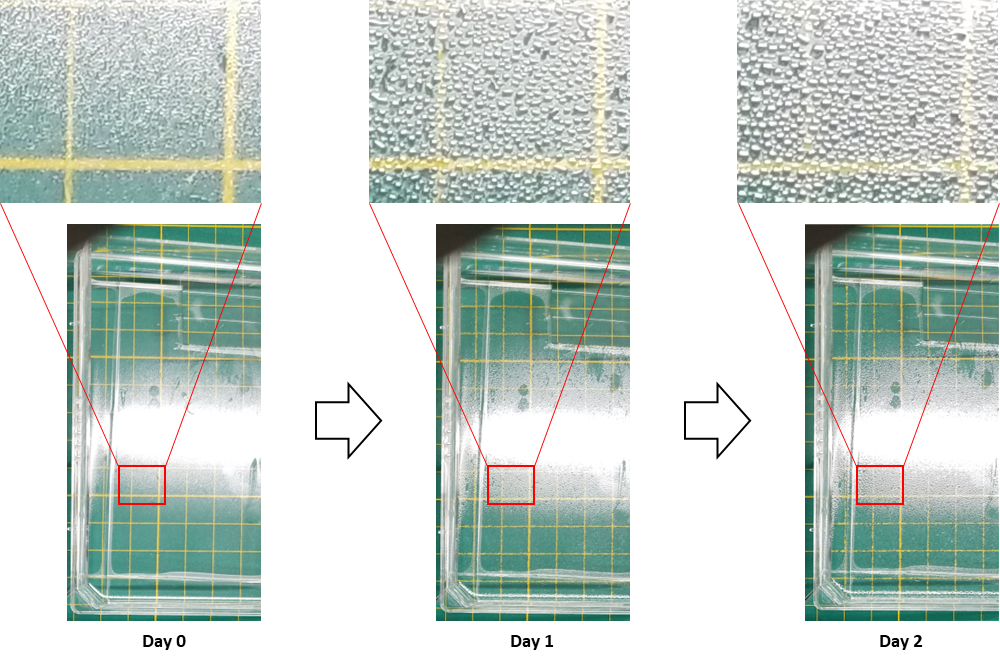


**Figure S8.** Photos of the oil-PDMS prepared by mixing PDMS (base:curing = 10:1 by weight) with silicone oil (base:oil = 5:1 by weight). Zoom-ups show that syneresis occurs and proceeds over time.


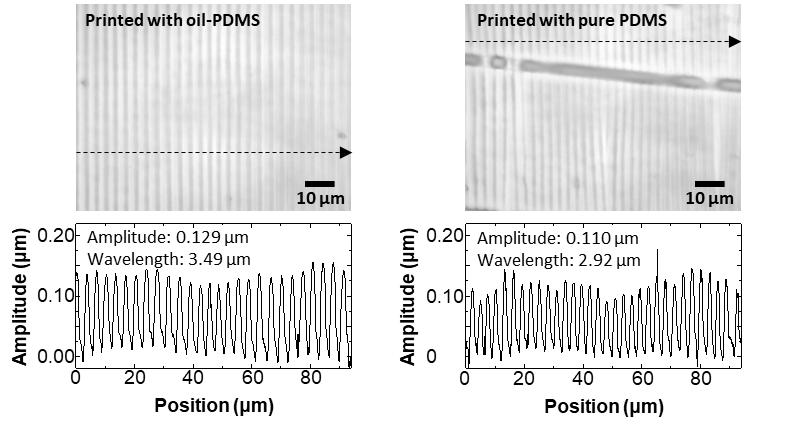


**Figure S9.** Laser microscope images and line profiles of the patterns printed using the oil-PDMS (left) and the pure PDMS (right). The line profiles are recorded along the dashed line arrow in the images above.

**Supplementary text**

To roughly estimate the possible number of printings using a single piece of 30:1-PDMS, we perform the following calculation by assuming that the cross-sectional shape of a printed structure is a simple triangle. All the parameters used for this calculation are listed in Table S1. Considering the dimensions of a 30:1-PDMS slab used in this study, its volume, $V_{PDMS}$, is given by

$V_{PDMS}=w_{PDMS}\cdot l_{PDMS}\cdot t_{PDMS}$,

where $w_{PDMS}$, $l_{PDMS}$, and $t_{PDMS}$ are the width, length, and thickness of the PDMS slab, respectively. Since some liquid PDMS is present in the PDMS slab with a weight ratio of $x$, the amount of liquid PDMS, $Q_{PDMS}$, is given by

$Q_{PDMS}=V_{PDMS}\cdot d_{PDMS\_L}\cdot x$,

where $d_{PDMS\_L}$ is the density of liquid PDMS and $x$ is the weight ratio of liquid PDMS residue in the PDMS slab. Assuming that a pattern consisting of stripes with a triangular cross-section is printed, the total amount of printed structure, $Q$, is

$Q=\frac{w\cdot l\cdot h}{2}\cdot\frac{l_{PDMS}}{w}\cdot d_{PDMS\_S}=\frac{l\cdot h\cdot l_{PDMS}\cdot d_{PDMS\_S}}{2}$,

where $w$, $l$, and $h$, are the width, length, and height of a printed structure, respectively, and $d_{PDMS\_S}$ is the experimentally obtained density of solid 30:1-PDMS. By dividing $Q_{PDMS}$ by $Q$, the possible number of printings using a single piece of 30:1-PDMS is estimated to be approximately 7,000.

**Table S1.** List of parameters used to estimate the possible number of printings using a single piece of 30:1-PDMS.

| Symbol | Detail | Value |
| --- | --- | --- |
| $w_{PDMS}$ | Width of a PDMS slab | 1 [cm] |
| $l_{PDMS}$ | Length of a PDMS slab | 3.5 [cm] |
| $t_{PDMS}$ | Thickness of a PDMS slab | 0.27 [cm] |
| $d_{PDMS\_L}$ | Density of liquid PDMS | 0.97 [g/cm^3^] |
| $d_{PDMS\_S}$ | Experimentally obtained density of solid 30:1-PDMS | 1.12 [g/cm^3^] |
| $x$ | Weight ratio of liquid PDMS residue in a solid PDMS slab | 0.15 |
| $w$ | Width of a printed structure | 0.00025 [cm] |
| $l$ | Length of a printed structure | 1 [cm] |
| $h$ | Height of a printed structure | 0.00001 [cm] |
